# Comparison of Engine Performance between Nano- and Microemulsions of Solketal Droplets Dispersed in Diesel Assisted by Microwave Irradiation

**DOI:** 10.3390/molecules24193497

**Published:** 2019-09-26

**Authors:** Cherng-Yuan Lin, Shih-Ming Tsai

**Affiliations:** Department of Marine Engineering, National Taiwan Ocean University, Keelung 202, Taiwan; trustme200266@yahoo.com.tw

**Keywords:** microwave irradiation, solketal, nanoemulsion, fuel property, engine performance.

## Abstract

As a derivative product of bio-glycerol, this study first uses solketal as a combustion improver for enhancing diesel engine characteristics. The emulsions of nanometer- and micrometer-sized droplets of solketal, which disperse evenly in the ultra-low sulfur diesel (ULSD), are formed by the effects of microwave irradiation. The performance of diesel engine fueled with the nanoemulsion of ULSD with scattered solketal droplets is analyzed and compared to that with the microemulsion. The experimental results show that the nanoemulsions can form when over 15 wt. % surfactant mixtures of Span 80 and Tween 80 and less than 5 wt. % solketal are mixed and emulsified with the remaining ULSD content, which acts as the continuous phase of the emulsions. The nanoemulsions are observed to have significantly lower brake-specific fuel consumption (bsfc) and higher fuel conversion efficiency and exhaust gas temperature than those of the microemulsions and the neat ULSD. However, the bsfc of the nanoemulsions increases with greater engine speed and gradually approaches those of the latter two test fuels. In addition, the dispersed solketal droplet sizes are mostly concentrated around 127 nm with peak intensity of 12.65% in the nanoemulsions. The microwave-assisted formation used in this study is found to successfully produce the nanoemulsions in which all of the dispersed droplet sizes are much smaller than 1000 nm.

## 1. Introduction

Glycerol acetonide, which is chemically derived from bio-glycerol through condensation reaction, is a colorless, transparent liquid. The global quantity of bio-glycerol has reached three million tons each year since 2015 due to the fast development of biofuels, particularly in Brazil, the U.S.A., and the European Union [1]. Glycerol acetonide, also termed solketal, with the chemical formula C_6_H_12_O_3_, has superior chemical stability and high oxygen content. The degree of complete burning of fossil fuel would be enhanced by adding such an oxygenate additive like MTBE or similar others, leading to the promotion of engine performance and the reduction of pollutant emissions [2,3]. In addition, a significant reduction of accumulating wax colloids in gasoline was observed after solketal was added to mix with gasoline [4]. Solketal also has been used as a fuel-fluidity improver to prevent fuel from freezing in frigid weather or low-temperature regions [5]. However, solketal has not been applied as a chemical additive of emulsion fuel to enhance engine characteristics. In addition, microwave irradiation has not been applied to enhance the formation of nano- or micro-emulsions of solketal in diesel as an engine fuel. Moreover, no study has been carried out to compare the effects of the physical nano- and micro-structures of diesel emulsions on diesel engine performance, although the emulsion structure might affect fuel stability and engine power output [6] in turn.

This study considers solketal as a diesel additive to improve the combustion characteristics of diesel engines fueled with emulsion fuel. The application potential of solketal in the energy field is evaluated as well. Diesel is not miscible with solketal due to the fact that the latter is a hydrophilic liquid. The emulsification method is thus applied to form homogeneous emulsions in which solketal is scattered to many droplets and dispersed evenly in the continuous diesel phase. After the emulsion fuel is atomized into liquid droplets by an atomizer, the solketal droplets in the interior phase, which own a lower boiling point than their outer enveloping diesel fuel, will vaporize and thereafter explode outwards through the continuous phase after the liquid solketal droplets absorb sufficient surrounding heat for evaporation. This is the so-called micro-explosion or second-atomization phenomenon of emulsion fuel [7]. Much smaller liquid droplets will be formed to increase the surface to volume (S/V) ratio of the atomized diesel emulsion so that the total contacting area and micro-explosion frequency between atomized liquid fuel and surrounding air are greatly raised [8]. Higher combustion efficiency might therefore result in [9]. Nanoemulsions with an average droplet size of dispersed phase lower than 1000 nm could also be produced after adding an adequate type and quantity of surfactant mixture [10]. Nanoemulsions exhibit optically transparent appearance due to the much weaker scattering strength of dispersed droplets exposed to the same projected light [11], the significantly higher thermodynamically stable fuel properties, and the much smaller droplets dispersed in the continuous phase, in comparison with those of microemulsions [12]. Mehta et al. [13] prepared water-in-diesel nanoemulsion together with nano-Al additive to improve the combustion characteristics of diesel fuel. Lin and Tsai [14] analyzed the emulsification characteristics of nanoemulsions of solketal in diesel and found the increase of solketal content in the emulsion caused the increase of the kinematic viscosity and mean droplet size and decrease of the emulsification stability. In addition, emulsion fuel has been used as alternative fuel of diesel engines to improve their engine performance [15].

Microwaves are electromagnetic waves, presenting characteristics of high frequency ranging from 300 MHz to 300 GHz and short wavelengths between 0.01m and 0.3 m [16]. Violent rotation of polar compounds occurs under the effect of microwave irradiation [17], resulting in fast friction among molecules and the temperature increase of the emulsion. Therefore, the dispersed droplets can be separated from their outer continuous phase. Hence, microwave irradiation has been previously applied for de-emulsification [18]. However, the high-frequency rotation of microwave irradiation meanwhile renders much higher contact frequency and mass transfer rate among various components of an emulsion [19]. In addition, energy loss during the transfer process and reaction time could be significantly reduced since microwave energy is transferred through radiation, resulting in superior heat transfer efficiency of microwave irradiation in comparison with that of regular hot-stir plate for heating a solution. Moreover, fast twist motion among the emulsion components impelled by microwave irradiation facilitates uniform distribution of dispersed phase within their continuous phase [20]. Hence, microwave irradiation could also be used to form the emulsion of various immiscible liquid phases under the effects of adequate irradiation energy [21,22]. This study thus applies microwave irradiation to prepare nanoemulsions and microemulsions of dispersed solketal droplets in ultra-low sulfur diesel (ULSD) by carefully controlling microwave irradiating power and time.

An emulsification method assisted by a microwave-irradiation reactor was used to enhance the homogeneity of these immiscible compounds and thus the reaction frequency among various phases. Various quantities of the surfactant mixture of Span 80 and Tween 80 were added to improve the emulsification stability of the diesel emulsions. The engine performances of microemulsions and nanoemulsions formed by the enhancement of microwave irradiation were analyzed and compared. The effects of the emulsion structure and engine speed on the engine characteristics were evaluated as well.

## 2. Experimental Details

### 2.1. Experimental Materials

Ultra-low sulfur diesel (ULSD) was supplied by CPC Corporation in Taiwan. The heating value, specific gravity (sg), cold filter plugging point (CFPP), flash point, carbon residue, and kinematic viscosity of ULSD were 46.26 MJ/kg, 0.83, −3 °C, 55 °C, 0.49 wt. %, and 3.62 mm^2^/s, respectively. ULSD was used as the continuous phase to encompass dispersed droplets of solketal in the nano- or microemulsions. Solketal played the role of combustion improver for ULSD in the form of a dispersed phase in the emulsions. The chemical formula, cold filter plugging point, specific gravity, kinematic viscosity, heating value, carbon residue, and flash point of solketal were C_6_H_12_O_3_, −4 °C, 1.06, 5.32 mm^2^/s, 14.99 MJ/kg, 1.81 wt. %, and 91 °C, respectively [23]. Hydrophile–lipophile balance (HLB) values of non-ionic surfactants Tween 80 and Span 80 were 15.0 and 4.3 [24], respectively, and were thus categorized to be hydrophilic, and lipophilic surfactants were provided by First Chemical Company in Taiwan. The chemical formula, flash point, heating value, and carbon residue of Span 80 were C_24_H_44_O_6_, 186.2 °C, 35.23 MJ/kg, and 2.87 wt. %, and C_64_H_124_O_26_, 148.9 °C, 29.77 MJ/kg, and 2.48 wt. %, respectively, for Tween 80 [24]. The surfactants were used to assist the formation of nano- or microemulsions through a reduction of interphase surface tension among various phases of the emulsions. The weight proportions of the surfactant mixture of Tween 80 and Span 80 were modulated to have a combined HLB value equal to 10.

### 2.2. Preparation of Nano- and Microemulsions by Microwave Irradiation

For preparing the nanoemulsion of solketal-in-ULSD, a mechanical homogenizer (T-50 model, Ika Inc., Staufen, Germany) was used to blend the ULSD with the surfactant mixture of Span 80 and Tween 80 homogeneously. The HLB value of the surfactant mixture was set at 10 by adequately adjusting the weight proportions of Span 80 and Tween 80, in which HLB was 4.3 and 15.0, respectively [24]. A peristaltic pump (MP-3, Eyela Inc., Tokyo, Japan) was then used to feed the solketal content to mix and stir with the ULSD and surfactant mixture in a beaker. The total volume of the entire mixture was 100 mL, consisting of 3 vol. % solketal, 15 vol. % surfactant, and ULSD the remaining. Lin and Tsai [25] observed the effects of microwave irradiation power and time on the temperature rise of three-phase emulsions of biodiesel-in-nitromethane-in-diesel (B/N/D). They found the temperature of the three-phase B/N/D emulsions was increased by 10 °C under microwave irradiation power of 0.15 kW for 2 min. Rao and McClements [26] inferred that stable multiple emulsions could be successfully formed under low emulsion temperatures below 50 °C. Moreover, the outmost continuous ULSD of the three-phase emulsion was not a polar compound. This implied that the rise of the multiple emulsion temperature and in turn the emulsion stability of such ULSD emulsion might be much less influenced by microwave irradiation. Hence, the power and time of the microwave irradiation was thus set at 0.1 kW and 30 s. to maintain the emulsion temperature rise below 3 °C in this study. The whole mixture in a beaker prepared in the previous stages was then moved into a microwave reactor (Ym3101cb model, Teco Inc., Taipei, Taiwan) to be irradiated at a power of 0.1 kW for 30 s. to complete the formation of the nanoemulsion of dispersed solketal-in-ULSD.

For preparing the microemulsions, 2 vol. % surfactant mixtures of Span 80 and Tween 80, instead of 15 vol. % surfactant mixtures for preparing the nanoemulsion as described above, in which the combined HLB value is equal to 10, was stirred with ULSD in a beaker by a mechanical homogenizer. Solketal of 3 vol. % was then fed into the beaker by a peristaltic pump and stirred to prepare a homogeneous mixture. A microwave reactor was used to irradiate the whole mixture in the beaker at an operating power of 0.1 kW for 30 s. to produce the microemulsion. After the nano- or microemulsions were prepared, the emulsion fuel was directly fed from the fuel tank through the high-pressure injection pump to the engine nozzles where the liquid fuel was atomized into fine spray and burned with surrounding air inside the combustion chamber of the diesel engine.

The distribution of the droplet sizes of dispersed phase in the nano- or microemulsion was analyzed by a particle size analyzer (Mastersizer 2000 model, Malvern Panalytical Ltd., Malvern, UK) equipped with a Zetasizer (Nano ZS model, Malvern Panalytical Ltd., Malvern, UK), adopting a dynamic light scattering (DLS) technique.

### 2.3. Measurement of Engine Performance

**Figure 1:**
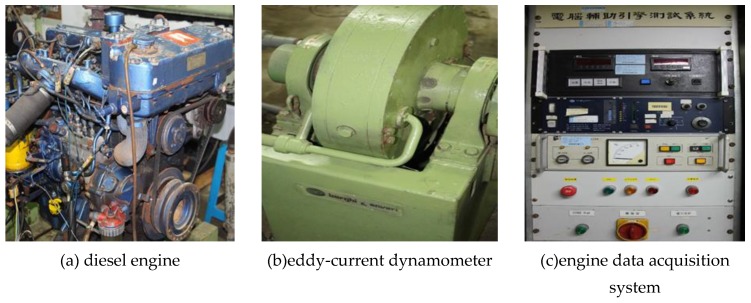
Photographs of the experimental equipment of (**a**) diesel engine, (**b**) eddy-current dynamometer, and (**c**) engine data acquisition system.

**Figure 2:**
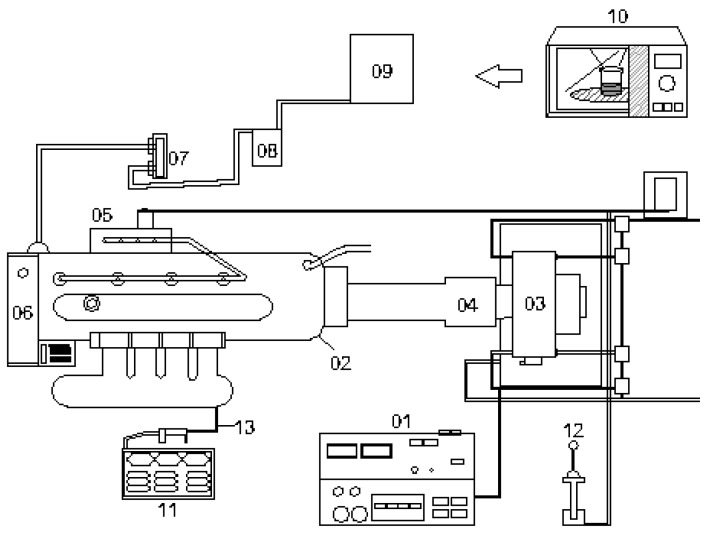
Set-up of diesel engine and dynamometer for analysis of engine performance fueled with diesel emulsions. Legends: (01) engine data acquisition system, (02) diesel engine, (03) dynamometer, (04) connecting shaft, (05) fuel pump, (06) engine radiator, (07) fuel meter, (08) fuel filter, (09) fuel tank, (10) microwave reactor for preparing test emulsion, (11) exhaust gas analyzer, (12) fuel throttle, and (13) exhaust gas probe.

A naturally air-aspiring, four-cylinder in-line, water-cooling, direct-injection diesel engine (UMBD1 model, Isuzu Inc., Tokyo, Japan) accompanied with an eddy-current dynamometer (FE-150S model, Borghi & Saveri Inc., Emilia-Romagna, Italy) was used to measure the engine performance of nano- and microemulsions of dispersed solketal-in-ULSD. The compression ratio, total displacement volume, and maximum output power of the engine were 17, 3856 cc, and 64.7 kW at 2800 rpm, respectively. The engine torque could be adjusted and controlled in the range between 0 and 176.4 N-m. The precision of the torque detector of the dynamometer was ±0.2%. The experimental engine data were collected and analyzed by an engine data acquisition system. The photographs of the experimental equipment including the diesel engine, eddy-current dynamometer, and engine data acquisition system are shown in Figure 1. The experimental set-up of the diesel engine and dynamometer for measurement of the engine performance fueled with the diesel emulsions is illustrated in Figure 2. The exhaust gas temperature was measured by a K-type thermocouple.

Brake specific fuel consumption (Bsfc) is defined as the fuel consumption rate required for the brake engine power output. A lower Bsfc value implies less fuel consumption to attain the same engine power output and thus is preferable. Bsfc is expressed as

(1) $$\mathrm{Bsfc}\;={\dot{m}}_{ƒ}/P,$$

where ${\dot{m}}_{ƒ}$ is the fuel consumption rate in unit of g/h, and P is the brake engine power output in unit of kW, which is the multiplication product of brake engine torque output and engine speed. Fuel conversion efficiency (η_f_) is the ratio of the brake engine work output corresponding to the amount of heat release from burning the engine fuel during an engine’s operating period. A higher η_f_ value indicates more engine power could be produced for the same mass of fuel burned. Thus, η_f_ is a reciprocal to Bsfc. The definition of η_f_ is expressed as

(2) $$\eta_{f}=\;\left(3600\;\left(\mathrm{kW}\right)\right)/\left(\mathrm{Bsfc}\;\left(g/\mathrm{kW}\cdot h\right)\;Q\_\mathrm{HV}\;\left(\mathrm{MJ}/\mathrm{kg}\right)\right),$$

where Q_HV_ is the heating value of the engine fuel. At least three repetitions in each experiment were carried out to obtain the mean values for the recorded data. The experimental uncertainties of mean droplet size of the dispersed phase, carbon residue, and heating value were ±2.36%, ±1.87, and ±1.05, respectively.

## 3. Results and Discussion

The engine performances of diesel engines fueled with the neat ULSD, micro-, and nanoemulsions of the ULSD were analyzed and compared. Solketal, also termed glycerol acetonide, played the role of a combustion improver, and was scattered into many micrometer- or nanometer-sized droplets and dispersed within the continuous phase of the ULSD. The experimental results are explained and discussed below.

### 3.1. Fuel-Structure Effects on Fuel Consumption Rate

The emulsions of solketal-in-diesel were prepared by microwave irradiation at a power of 0.1 kW for 30 s. Microwave irradiation caused fast and violent friction among the emulsion components, resulting in temperture rise of the emulsion. The temperatures of the nano- and microemulsions were observed to increase by 2.8 °C and 2.3 °C, respectively. The slightly larger temperature rise of the former emulsion might be attributed to larger total contacting area of nanometer-sized droplets of solketal, which cause more violent friction under the effect of microwave irradiation.

Three test fuels with various physical structures, including the neat ULSD, microemulsions, and nanoemulsions of dispersed solketal-in-ULSD, were considered. The properties of components in the nano- or microemulsions, including solketal and the surfactants Span 80 and Tween are compared in Table 1 [23,24]. The dispersed phase of solketal was shown to have the lowest amounts of heat release and carbon residue among those components of the nano- or microemulsions. Lower carbon residue indicates a more extent of complete combustion and thus solketal could be an adequate oxygenate additive to enhance fuel burning. However, the higher weight fraction of solketal in the emulsions would reduce the amounts of heat release from the combustion of those emulsions [27]. In contrast, the surfactants Span 80 and Tween 80 are composed of energy dense molecules and high content of molecular oxygen. The weight percentage of oxygen content of solketal is 36.36%, which is somewhat higher than those of Span 80 and Tween 80, which are 31.80% and 22.43%, respectively. Higher oxygen content in fuel would enhance more complete fuel burning and less carbon residue left in turn. In addition, the amounts of heat release of Span 80 and Tween 80 are 1.99 and 2.35 times of that of solketal. Lin and Tsai [14] compared the emulsification characteristics of solketal-in-ULSD emulsions prepared with surfactant mixture of Span 80 and Tween 80 ranging from 5 wt. % to 20 wt. % and solketal ranging from 1 wt. % to 9 wt. % by microwave irradiation. They found that the increase of the weight fraction of the surfactant mixture along with the decrease of solketal content in the emulsions resulted in higher amounts of heat release and lower mean droplet sizes. However, the kinematic viscosity of the emulsions increased with the increase of the weight proportion of the surfactant mixture due to more viscous surfactant mixture than other components in the emulsions, resulting in atomization difficulty, discontinous buring of the emulsion fuel, and higher carbon residue in turn [28].

The representative properties of those fuels in which engine performance was measured are listed in Table 2. The neat ULSD was shown to have the highest amount of heat release (46.26 MJ/kg), while the microemulsion was the lowest one (43.58 MJ/kg) among those fuels. The mean droplet size of the nanoemulsion was only 0.13 µm, which was only about 0.5% of that size of the microemulsion (26.51 µm). The total surface area among various phases of components of the nanoemulsion was thus significantly larger than the other two fuels, resulting in it being the largest kinematic viscosity among the test fuels. The result agreed well with Cabaleiro et al. [29]. The nanoemulsion was also found to have the lowest carbon residue (0.26 wt. %), which indicated the greatest extent of complete reaction to have the least unburnt fraction of liquid fuel. The physcial structure of dispersed droplets of nano-metered sizes distributed in the continuous phase of ULSD, formed by assistance of microwave irradiation, greatly enhanced the extent of chemical reaction and stabililities of thermodynamics and emulsification of the nanoemulsion. An emulsion with smaller dispersed droplets was found to have superior phase stability, atomization, and burning characteristics, leading to less carbon residue left after burning [25].

**Figure 3:**
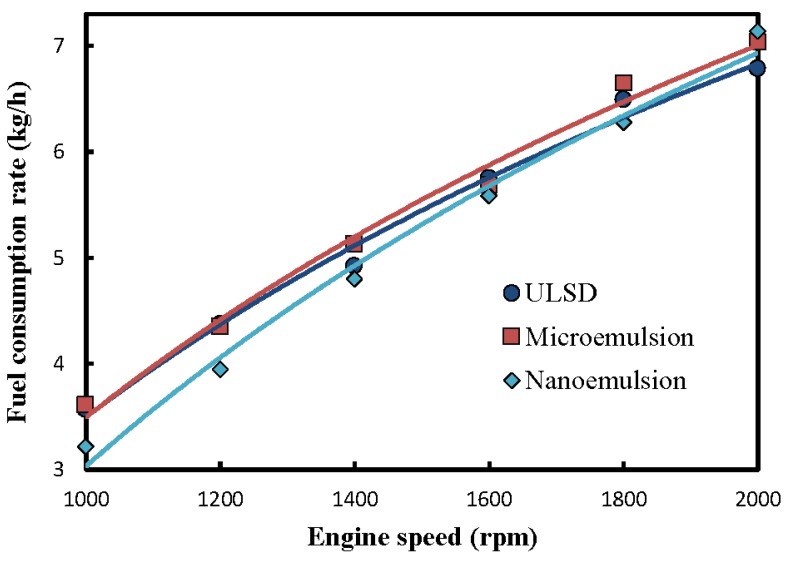
Effects of fuel structure and engine speed on the fuel consumption rate of a diesel engine.

The effects of various physical structures of the test fuels including the neat ULSD, micro-, and nanoemulsions of dispersed solketal-in-ULSD on the fuel consumption rate are shown in Figure 3. Both the nano- and microemulsions of solketal-in-ULSD released less heat than the neat ULSD due to the lower heating value of solketal (14.99 MJ/kg), Span 80 (35.23 MJ/kg), and Tween 80, (29.77 MJ/kg) than the ULSD (46.26 MJ/kg). The data of original fuel consumption rate of both the micro- and nanoemulsions were corrected by multipying such originally acquired data by the ratio of the heating values of the ULSD to either the micro- or nanoemulsion. Hence, all the three different fuels considered here have the same amount of heat release as that of the ULSD under the preset engine operating condition after correcting for the heating values of ULSD and solketal.

The fuel consumption rates of various fuels are observed in Figure 3 to increase with greater engine speed. The engine power output increased with a rise in engine speed under unvaried engine torque (147 N-m). A higher fuel consumption rate was required to achieve higher engine power output [30]. Moreover, the friction horsepower generally increased with higher engine speed. A higher fuel consumption rate was thus necessary at higher engine speed.

The nanoemulsion was found to have the lowest fuel onsumption rate, particularly at engine speeds lower than 1800 rpm. A larger number of nanometer-sized dispersed droplets of solketal, which was more uniformly distributed within the continuous phase of the ULSD, could have promoted the physical and thermodynamic stabilities of the nanoemulsion. A larger extent of secondary atomization therefore occurred, leading to higher combustion efficiency for the nanoemulsion. The photographs of optical electron microscope associated with a charge-coupled device provided by Lin and Tsai [14] illustrated the dispersed droplet distribution of the solketal-in-diesel nanoemulsion after being prepared by microwave irradiation and kept motionless for 7 days. They found thermodynamically stable nanoemulsion in which nanometer-sized droplets were well-distributed in the continuous diesel phase. Hence, a lower fuel consumption rate was required for the diesel engine fueled with the nanoemulsion in Figure 3. However, the increased engine speed in turn caused greater engine power output and production of higher thermal energy. The physical structure of the emulsion was therefore destroyed by the high engine operating temperature to some extent. Dispersed solketal droplets thereafter precipated from their emulsification layer, resulting in a lower amount of heat release from burning the emulsion at the higher engine speed. Hence, the fuel consumption rates of the emulsions began to exceed that of ULSD after the engine speed was increased to beyond 2000 rpm, as shown in Figure 3.

### 3.2. Fuel-Structure Effects on Brake Specific Fuel Consumption (Bsfc)

**Figure 4:**
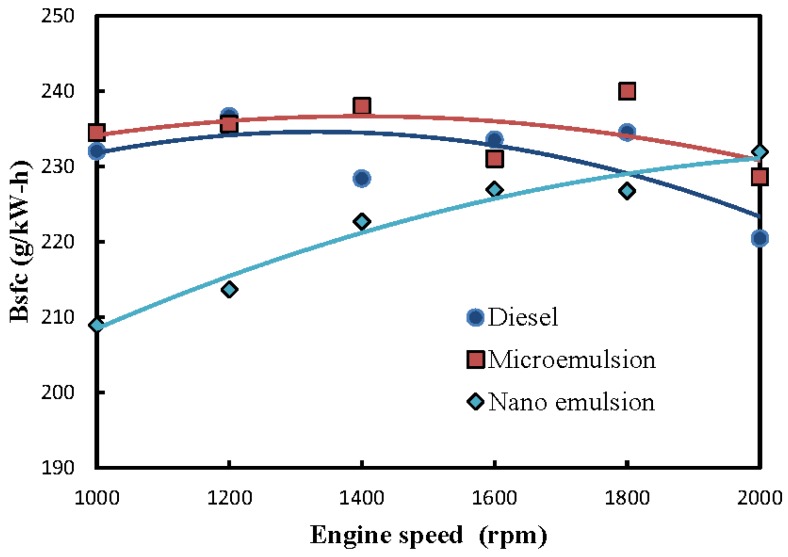
Fuel structure and engine speed of brake-specific fuel consumption (Bsfc) of a diesel engine.

Brake-specific fuel consumption (Bsfc) is defined as the fuel consumption rate corresponding to the production of the brake engine power output in Eq. (1). Bsfc could be used as an indicator of conversion efficiency from chemical energy to thermal energy for various engine fuels. Bsfc could also be applied to compare operating efficiency between different engines fueled by the same fuel. A lower Bsfc value indicates superior fuel conversion efficiency or engine operating efficiency and is therefore preferrable [31]. The Bsfcs of both the microemulsion and neat ULSD were observed to decrease with an increase of engine speed in Figure 4. Moreover, the Bsfc of the microemulsions was larger than that of the ULSD. It is ascribed to the physical structure of the microemulsion being somewhat destroyed by the high operating temperature, particularly at high engine speeds, so that a greater mass fuel flow rate was required to attain the same engine power output as the ULSD.

The nanoemulsions were shown to have the lowest Bsfc or, equivalently, the highest energy conversion ratio from chemical energy to thermal energy under engine speeds below 1800 rpm. However, the Bsfc increased with a rise in engine speed, which indicated a deteriorating fuel conversion rate at faster engine speeds. It is ascribed to the considerably higher kinematic viscosity (7.2 mm^2^/s) of the nanoemuslion and the decreased fuel conversion rate under increasing engine speeds. The experimental results of Venkatesh et al. [32] also showed that the increase of kinematic viscosity of nanofluid caused the rise of specific fuel consumption. An increasing fuel consumption rate with a higher engine speed was thus required, as shown in Figure 3. Hence, the Bsfc of nanoemulsion was observed to increase more significantly with an increased engine speed, particularly at the higher engine speed in Figure 4.

### 3.3. Fuel-Structure Effects on Fuel Conversion Efficiency

Fuel conversion efficiency is defined as the ratio of engine power output produced from the corresponding fuel consumption rate for an engine based on Eq. (2). A higher fuel conversion rate indicates a higher conversion extent of fuel chemical energy to engine output work and therefore is more preferrable. Hence, a higher Bsfc implies a lower fuel conversion efficiency for the same fuel.

**Figure 5:**
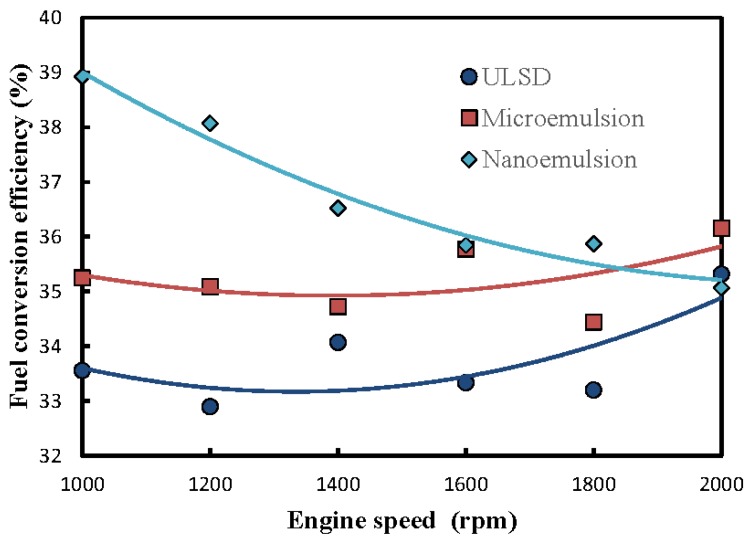
Fuel structure and engine speed of fuel conversion efficiency (%) of a diesel engine.

The effects of emulsion structure and engine speed on fuel conversion efficiency are shown in Figure 5. Both the nano- and microemulsions appeared to have higher fuel conversion efficiency than the ULSD. In addition, the nanoemulsion of dispersed solketal droplets in ULSD was observed to have the highest fuel conversion efficiency among those three test fuels. This is primarily because of the combustion enhancement of solketal, which contains as high as 36.36 wt. % oxygen. Moreover, higher extents of homogeneous and complete burning might occur for the nanoemulsion in an engine combustor due to its larger contacting areas among the components of reactant mixture [33]. It was also found that the fuel conversion efficiencies of the microemulsion and the neat ULSD fuel increased slightly with the engine speed in Figure 5. This implies that the fuel conversion efficiencies of those two fuels improved at higher operating temperatures produced under higher engine speeds. In contrast, the nanoemulsion was found to have decreased fuel conversion efficiency with the increase of engine speed. This is ascribed to the decreasing degree of complete reaction when the more viscous nanoemulsion, with kinematic viscosity of 7.2 mm^2^/s or about twice that of the other two test fuels, was burned in a relatively shorter reaction time at a higher engine speed. Hence, a higher fuel consumption rate was necessitated to compensate for such a lower burning extent, leading to the decreased fuel conversion efficiencies with increased engine speeds for the nanoemulsion in Figure 5.

### 3.4. Fuel-Structure Effects on Exhasust Gas Temperature

**Figure 6:**
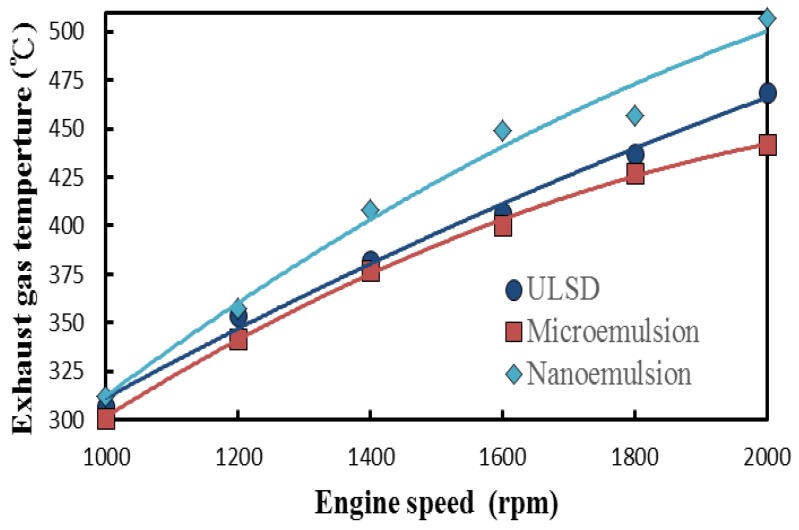
Effects of fuel structure and engine speed on exhaust gas temperature (°C) of a diesel engine.

The exhasust gas temperatures of the diesel engine fueled with the nano- and microemulsions and ULSD under various engine speeds are shown in Figure 6. All the test fuels were found to have increased exhaust gas temperature with greater engine speed. This is due to the increased engine power output with the rise of engine speed under unvaried engine torque, in turn leading to higher fuel consumption rates and exhaust gas temperatures for the test fuels. Moreover, the nanoemulsions had the highest exhaust gas temperatures and the largest increase rate of exhaust gas temperature with the increase of engine speed among the three aforementioned test fuels. In comparison with that of the microemulsion, many more tiny dispersed solketal droplets within the continuous ULSD phase of the nanoemulsion increased both the total surface to volume (S/V) ratio of the fuel spray and reacting area with surrounding air. This was followed by a more complete burning, higher amount of heat release, and thus higher exhaust gas temperature for the nanoemulsion. Hence, Bidita et al. [34] inferred that nanoemulsion fluid is mostly used in various applications including energy, pharmaceutics, and chemistry fields.

The microemulsion of solketal-in-ULSD was observed to have the lowest exhaust gas temperature. The physical structure of the microemulsion was more susceptible to be broken at high operating temperatures produced under high engine speeds due to inferior emulsification stability [35] than by that of the nanoemulsion. The dispersed solketal droplets in the microemulsion were not enveloped completely by their continuous ULSD phase, so that the degree of micro-explosion for enhancing the combustion extent was reduced. A lower amount of heat release and thereafter lower exhaust gas temperature thus appeared in Figure 6 for the microemulsion.

### 3.5. Fuel-Structure Effects on Distribution of Dispersed Droplet Size

**Figure 7:**
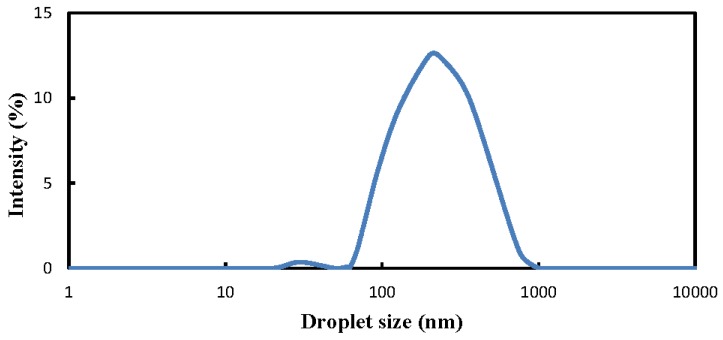
Distribution of dispersed droplet size of solketal in the continuous diesel phase of nanoemulsion with 3 wt. % solketal used for the engine test.

The size distribution of the dispersed solketal droplets of the nanoemulsion of ULSD for fueling the diesel engine, which was analyzed by a particle size analyzer (Master 2000 model, Malvern Panalytical Ltd., Malvern, UK) accompanied with a Zetasizer (Nano ZS model, Malvern, Panalytical Ltd., Malvern, UK), is shown in Figure 7. The droplet sizes of the nanoemulsion were found to be mostly concentrated around 127 nm, with a peak intensity of 12.65 %, as shown in Figure 7. Moreover, all the sizes of the dispersed solketal droplets of the nanoemulsion were smaller than 1000 nm. This implies that the nanoemulsions of solketal-in-ULSD for fueling the diesel engine were successfully produced by the microwave irradiating effects in this study.

## 4. Conclusions

This study experimentally investigated the engine performance of a diesel engine fueled with micro- and nanoemulsions of solketal droplets dispersed within the continuous phase of the ultra-low sulfur diesel (ULSD). The main results of this study are summarized below.

(1) The nanoemulsions of solketal-in-ULSD had the highest kinematic viscosity, lowest mean droplet size of the dispersed phase, the lowest carbon residue, and the lowest fuel consumption rate required for the same engine power output among the three test fuels. In addition, a larger fuel consumption rate was required at higher engine speed for all the test fuels;
(2) In comparison with microemulsions of solketal droplets in ULSD and the neat ULSD, the nanoemulsions were found to have the lowest brake specific fuel consumption (Bsfc) and equvilently the highest fuel conversion efficiency. This implies that the nanoemulsions of solketal-in-ULSD had superior fuel economy and a lower fuel consumption rate and are thus preferrable to fuel diesel engines among those three fuels. In addition, the Bsfcs of the nanoemulsions increased while fuel conversion efficiency decreased under an increase of engine speed. The Bsfc and fuel conversion efficiency of the nanoemulsion gradually approached those bsfcs and fuel conversion efficiencies of the microemulsions and the neat ULSD particularly at a faster engine speed of over 1600 rpm;
(3) The nanoemulsions of the dispersed solketal droplets within the ULSD were observed to have the highest amount of heat release and the highest exhaust gas temperature among those three test fuels;
(4) Most of the dispersed solketal droplet sizes were concentrated around 127 nm, with peak intensity of 12.65%. In addition, the microwave-irradiating effects applied in this study were successful in preparing the nanoemulsions in which all the sizes of the dispersed solketal droplets distributed within their continuous ULSD phase were well below 1000 nm.

## Figures and Tables

**Table 1:** Properties of the emulsion components [23,24].

Property	Component		
	Solketal	Tween 80	Span 80
Chemical formula	C_6_H_12_O_3_	C_64_H_124_O_26_	C_24_H_44_O_6_
Molecular weight (g/mol)	132	1308	428
Flash point (°C)	91	149	186
Amount of heat release (MJ/kg)	14.99	29.77	35.23
Carbon residue (wt. %)	1.81	2.48	2.87

**Table 2:** Fuel properties of engine test fuel.

Property	Fuel		
	Neat ULSD	Microemulsion of Solketal-in- ULSD	Nanoemulsion of Solketal-in- ULSD
Amount of heat release (MJ/kg)	46.26	43.58	44.26
Carbon residue (wt. %)	1.05	0.29	0.26
Kinematic viscosity (mm^2^/s)	3.4	3.6	7.2
Flash point (°C)	79	84	87
Mean droplet size (µm)	-	26.51	0.13
